# Daily Activity Patterns of Two Co-Occurring Tropical Satyrine Butterflies

**DOI:** 10.1673/031.009.5401

**Published:** 2009-07-14

**Authors:** Paulo Enrique Cardoso Peixoto, Woodruff W. Benson

**Affiliations:** Institutional Affiliation: Department of Zoology, lB, State University of Campinas, São Paulo, Brazil, C.P. 6109, 13083-970

**Keywords:** Satyrinae, mating tactics, territoriality, daily activity pattern, mate location

## Abstract

Adult males and females of many insect species are expected to adjust their daily activity pattern in order to avoid stressful climatic conditions and increase the chances to encounter sexual partners. Using scan sampling methods associated with focal individual observations it was found that two satyrine butterflies of similar size and morphology, *Hermeuptychia hermes* (Fabricius) (Leptidoptera: Nymphalidae) and *Paryphthimoides phronius* (Godart), show completely different daily activity patterns on forest edges in southeastern Brazil. *Hermeuptychia hermes* presents one abundance peak in the morning and another in the late afternoon, while *P. phronius* abundance peaks in the mid-day, remaining stable until 1700 h. This difference is probably due to the occurrence of territorial behavior in the later species. The beginning of territorial defense by *P. phronius* males coincided with the time of new-born female activity. However, newly hatched females were not sexually receptive. The afternoon territoriality in male *P. phronius* may be in part related to mate acquisition. However, why the abundance of *H. hermes* decreases when the abundance of *P. phronius* increases is less clear.

## Introduction

In continuously reproducing polygamous animals, such as many tropical butterflies, male fitness is expected to be directly proportional to its mating success, and striving after mating may permeate the activity schedule of males during the day (Rutowski 1984; Heinrich 1986; Rutowski 1991). Female butterflies, on the other hand, store sperm, and after mating once, (or a few times in some species), reproductive success may depend primarily on the careful distribution of eggs in places where larvae can survive. To maximize individual fitness, the daily activity pattern of both males and females are expected to adjust adaptively by reducing the impact of unfavorable abiotic conditions, the difficulties in acquiring resources and sexual partners, and the risk of falling victim to natural enemies.

Daily variations of solar radiation, temperature, wind and humidity may have profound effects on when an insect should be active and what it should be doing at any given time. Abiotic constraints may be especially important to small ectotherms such as butterflies because of their reduced thermal inertia (*sensu* Rutowski 1994). In particular, daily variations of solar radiation and air temperature may strongly influence the activity pattern and the types of habitat used by small species due to their great surface/mass ratio (May 1979). Therefore, periods of high air temperatures and solar heating may be potentially lethal (Rawlins 1980; van der Have 2002), and heat, even when not permanently debilitating, may aggravate desiccation, compromise adaptive behavior (Rutowski et al. 1994; Ide 2002b) and impose unacceptable metabolic costs (Renault et al. 2003; Terblanche et al. 2005). On the other hand, when temperatures are too low, oviposition and mate search may be inefficient and predator escape compromised (Srygley and Kingsolver 2000; Berwaerts and Van Dyck 2004).

Butterflies have many ways to regulate body temperature and avoid stressful conditions during the day by altering posture or changing microhabitat (Clench 1966; Rutowski et al. 1994; Ide 2002a). In this sense, butterfly abundance may reflect activity since sunny areas such as forest edges or sunspots may be preferentially occupied during periods of low to moderate temperatures but avoided when temperatures are too high. On the other hand, the time of day during which females become available to mate, may also play an overriding role in the daily activity patterns of male butterflies (Kemp and Rutowski 2001; Hirota et al. 2001). When females are immediately receptive after emergence and their location is predictable, males may adjust their search for sexually receptive partners to periods and places of high female availability (Deinert et al. 1994; Hernández and Benson 1998; Hirota et al. 2001). However, species in which females can postpone mating to a day or more after emergence may become increasingly able to determine the period, location and quality of successful males (Kemp and Rutowski 2001). Hence, male butterflies are sometimes expected to face a tradeoff between the need to find sexually receptive females and potentially restrictive climatic conditions.

Despite the striking changes in butterfly activities during the day (Clench 1966; Lederhouse 1982; Heinrich 1986; Hirota et al. 2001; Kemp and Rutowski 2001), daily activity patterns have rarely been studied in non-social insects (but see Morewood et al. 2004). Thereby, little information exists for insects such as butterflies on the timing and intensity of essential activities, as well as on the kinds of factors and tradeoffs that might structure their activity schedule. Although daily activity patterns have sometimes been reported in butterflies (Braby 1995; Hirota and Obara 2000a; Hirota and Obara 2000b; Hirota et. al. 2001), few studies have been designed to describe and understand how and why organisms partition activities during the day.

Studies on heliotherms, (i.e., those organisms that obtain heat from the sun) seem especially useful because of the expected strong responses to microclimate. In this sense, it is also important to account for climatic variations on different scales due to the possible interaction between microclimate and environmental characteristics on heliotherm responses (Kemp and Rutowski 2001). Especially if the goal is to compare daily activity schedules of closely related species one must be able to disentangle the responses related to climatic variations from the responses related to habitat differences in the area of occurrence. In this situation, comparative studies using species that live in the same place seem to be especially useful to understand how incompatible needs impinge on organisms over time and how conflicts are accommodated with regard to predictably changing conditions over the day.

Here we investigate the daily activity pattern in co-occurring populations of two previously unstudied species: *Hermeuptychia hermes* (Fabricius) and *Paryphthimoides phronius* (Godart) (Lepitoptera: Nymphalidae: Satyrinae: Euptychiina). The objectives were two-fold: first, the daily abundance pattern (used as a surrogate of activity) of both species is described, analyzing the possible factors that directly and indirectly influence their daily abundance variations. Second, a detailed description of behavioral variations during the day for *P. phronius* males is presented and the role of female emergency schedule in response to male behaviors related to mate-acquisition is investigated. The expectation is that, due to their morphological similarities (see below), *P. phronius* and *H. hermes* will become more active and more abundant on the forest edge during periods of low to moderate temperature. For *P. phronius* it is also expected that males may become more active when female availability is high.

## Materials and Methods

### Study area

The study was carried out in the Santa Genebra Municipal reserve (22° 50″ S, 47° 06′30″ W), Campinas, São Paulo state, southeastern Brazil. The reserve is a subtropical semideciduous forest fragment of 250 ha surrounded by farmland and residential areas (Galetti 1993). The local climate is seasonal, with a cooler, dry period from May to August and a warmer, rainy period between November and February (Galetti 1993). The mean temperature registered along the day during the study period was very similar between seasons: 25° C during the dry season and 26° C during the rainy season. However, the temperature in the dry season was much more variable (SE = 2.41° C) when compared to the rainy season (SE = 0.71 ° C).

The study was conducted on two adjacent areas of forest edge along the border of the reserve. The forest edge was surrounded by an unpaved one-lane access road that circled the reserve just inside a protective wire fence. The two areas, one 150 m, long and the second 300 m, were selected on the basis of differences in the presence of vegetation on the margin protected by the wire fence. Each area was subdivided into 3 × 4m parcels by marking the fence along the edge of the reserve, which were used as sample units to count butterflies.

**Figure 1.**
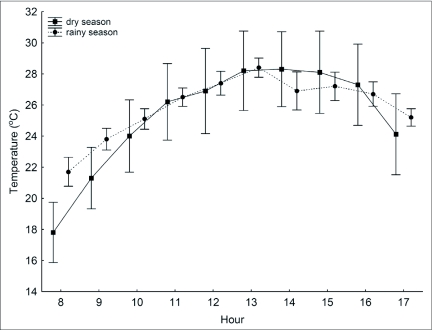
Daily variations of temperature on the dry and the rainy seasons averaged across all areas and 5 sampling days per season. Bars indicate standard error.

Area 1 was divided into 50 parcels (1 to 50). Here a paved road outside the reserve was parallel to the fence. The margin delimited by the fence had a few trees and grass that were frequently cut. In area 2, which began 20 m beyond area 1, the fence was accompanied outside the reserve by a wooded strip 3–5 m wide. In each area, the access road (approximately 3 m wide) was bordered internally by forest edge, usually with grasses and varying mixtures of herbs and shrubs in a band varying from a few centimeters to approximately 4 m in width.

### Study organisms

*Paryphthimoides phronius* and *H. hermes* are small, often abundant, and found in pastures, forests margins and disturbed woodland across much of tropical South America (Ramos 2000; Motta 2002). See Peña et al. (2006) for a review of satyrine phylogeny and generic placement. Both butterflies are nondescript dull brown and fly low among weeds and bushes. Larvae feed on grasses (Poaceae) and adults on rotting fruit and plant exudates. The most conspicuous biological differences in the adults are the slightly larger size of *P. phronius* (mean wing length= 17.7 mm; mean weight = 23 mg; n = 25) and its black eyes when compared to the brown eyes of *H. hermes* (mean wing length = 17.3 mm; 22 mg; n = 25).

**Table 1.**
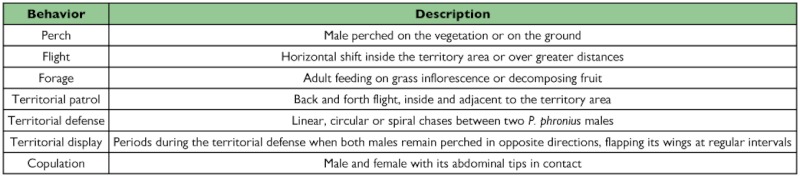
Behavioral ethogram of *Paryphthimoides phronius* males

### Daily abundance pattern

Daily general butterfly activity was measured by counting individuals of *P. phronius* and *H. hermes* seen in each 3-m parcels during hourly transects through the two areas. For *H. hermes*, males could not be distinguished from females, but for *P. phronius*, each sex could be distinguished based on size differences and flying pattern. Females were visibly bigger than males and typically presented very brief flights, usually inside areas with abundant grass or through the underbrush. One of us (PECP) conducted scans from 8 h to 17 h (local standard time) on 5 days in the rainy season and 5 days in the dry season (there were from 5 to 29 days of interval between adjacent scans to reduce temporal dependence between counts). All scans were conducted on sunny days, which were started at the beginning of each hour and took approximately 20 to 30 minutes (depending on the number of individuals) to complete butterfly counting through the 450 m study area. Air temperature and relative humidity were measured with a sling Psychrometer at the beginning of each transect. Comparisons of the daily activity schedule between climatic variables and the total counts of *P. phronius* and *H. hermes* were done using a repeated measures analyzes of variance (the original data met the sphericity assumption and thus were not transformed Quinn and Keough 2002). The total number of individuals was used in each hour as the response variable and species as the predictor variable. Day was used as the sample unit.

Abundance and climate data for each hourly sample were averaged over the five sampling days for each area and season. An analysis of covariance was used to investigate correlations between air temperature and butterfly activity (transect counts) across the day. Butterfly abundance (mean number of individuals per hour averaged across five days) was considered as the response variable and temperature and season as the independent variables (Quinn and Keough 2002). Relative humidity measured on each hour and sample day was strongly correlated with temperature on the dry (Linear regression, b= -0.52, F_(1,48)_=53.74, r^2^=0.52, p<0.001) and the rainy (Linear regression, b=-0.35, F_(1,48)_=54.83, r^2^=0.52, p<0.001) seasons (Figure 1). Because of the strong association between the two variables and the judgment that humidity was potentially less important than temperature as a stress factor, humidity was excluded from analyzes.

### Daily behavioral pattern of *P. phronius* males

Between successive count transects, randomly chosen *P. phronius* males were observed for 30 min with the aid of a mini cassette-recorder and the time it spent in each activity was calculated (Table 1). Focal observations were restricted to males because mate search is concentrated in exposed places where observation is relatively easy, whereas females were not common on the study area and, when present, occur on the underbrush where they are difficult to keep in view.

It was not always possible to follow an individual during 30 consecutive minutes. The exclusion of these observations from analyses could substantially reduce the number of sampled males and would introduce a bias because more active males were probably more difficult to follow for the total observation period. Thus, we opted to include in the analyses individuals that were followed for less than 30 min, and, in order to reduce the differences in the observation time among males, the proportion of time spent in each behavior was used.

### Behavior of sexually receptive *P. phronius* females

In order to investigate the influence of sexually receptive females on the activity pattern of male *P. phronius*, females were reared from eggs in the laboratory and released in the field. Eggs were obtained from field-caught gravid females induced to oviposit by holding them in plastic bags under a desk lamp. Larvae were raised separately in 250 ml plastic bottles maintained near a window at room temperature. The bottles were cleaned every day, and larvae were fed twice a day with fresh cuttings from the host-plant *Axonopus compressus* (Swarts 1812) Beauv. (Poales: Poaceae).

**Table 2.**
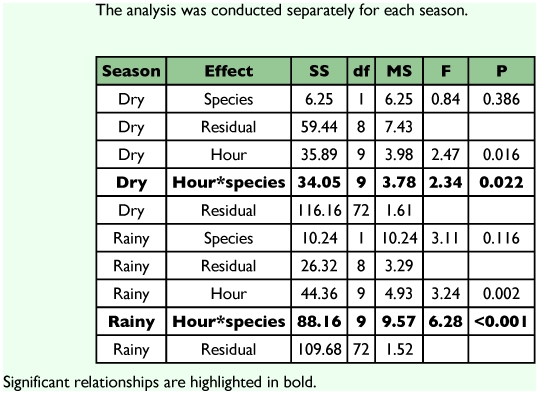
Repeated measures ANOVA comparing total counts of *Paryphthimoides phronius* and *Hermeuptychia hermes* over the day in the area 1.

All individuals were weighed to the nearest milligram on the day following pupation. Females were clearly separable from males based on the pupal weight before emergence. From a total of 27 samples in a pilot study, all males (n = 15) emerged from pupae with 63 to 73 mg and all females emerged with 83 to 103 mg (n = 12). There were no pupae with 74 to 82 mg and therefore no weight overlap between the sexes. Moreover, it was possible to predict the day of emergence because all pupae changed color when adults were ready to emerge. Hence, pupae were placed in the field on the day of emergence to avoid possible female behavioral changes caused by hand manipulation of adults.

The hour of female emergence was measured as the pupal skin split. Female pupae were allowed to emerge “naturally” a short distance (1 to 3 m) from places frequented by adult males. All behavioral or morphological changes were timed after pupal skin split and these females were observed for 1 h after their first spontaneous flight or until they were lost from sight.

In addition, in order to investigate possible delays in the female sexual receptivity, other laboratory-raised females, one to four days of age, were observed in the field by carefully releasing them on low vegetation near territorial males. Using a black marking pen, the forewings of these older females were marked to permit their visual identification without recapture. After releasing, the behavior of these females was observed following the same protocol used for newborn females.

## Results

### Daily abundance pattern

**Figure 2.**
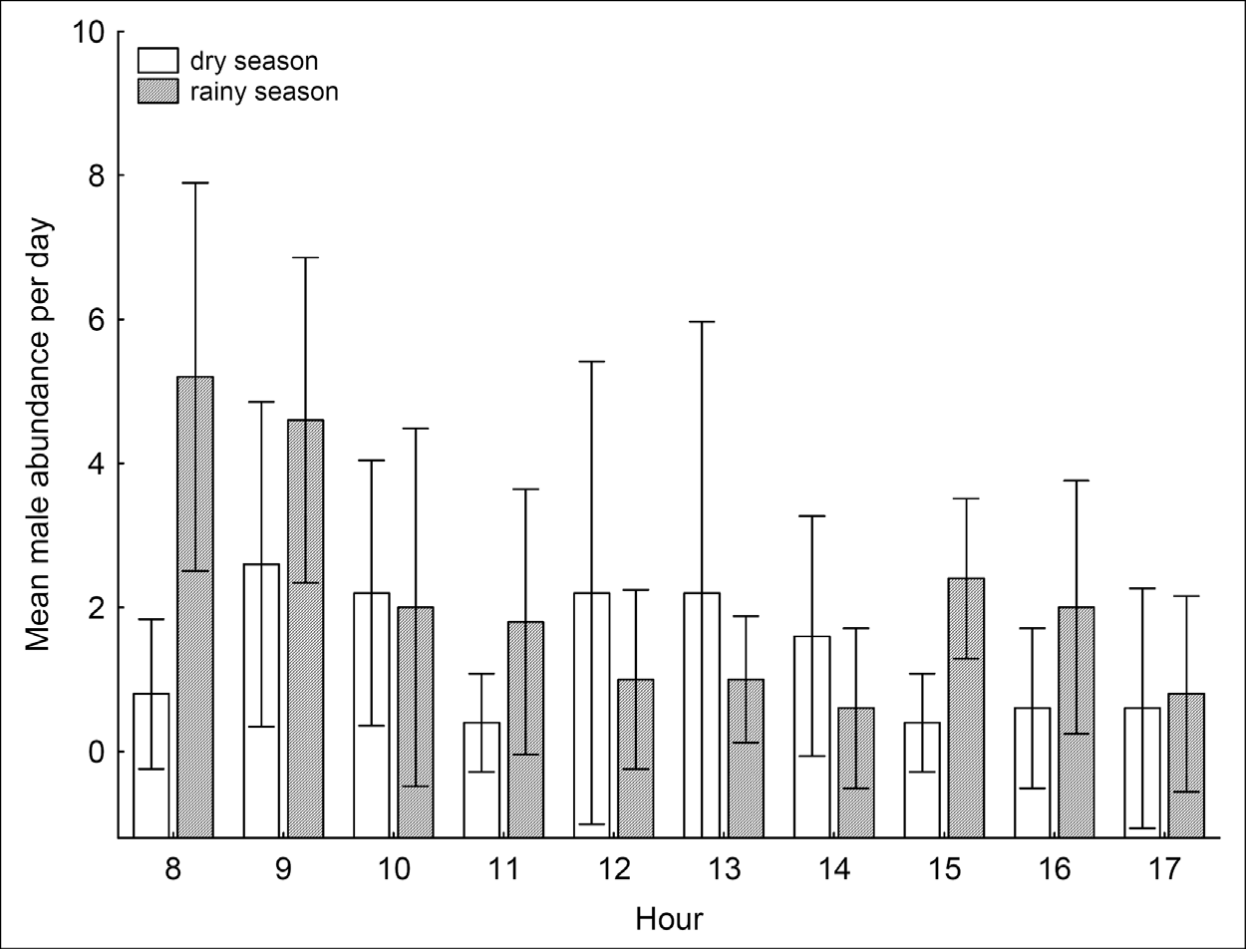
Variation in the abundance of *Hermeuptychia hermes* along forest edge transects taken hourly over the day. Bars represent the 95% confidence interval for means of total count based on samples from 5 days.

*Hermeuptychia hermes* was found only in area 1 and, although numerically similar to *P. phronius*, seemed to differ in the levels of activity during the day in dry and the rainy seasons (Table 2). *Hermeuptychia hermes* has two daily abundance peaks, which are especially evident in the wet season when one peak occurred early, from 0800 to 0900 h, and the other in the afternoon at 1500 h (Figure 2). During the dry season, the peaks were nearer to midday (0900–1000 h and 1200–1400 h), and were separated by a brief but marked low activity around 1100 h.

**Figure 3.**
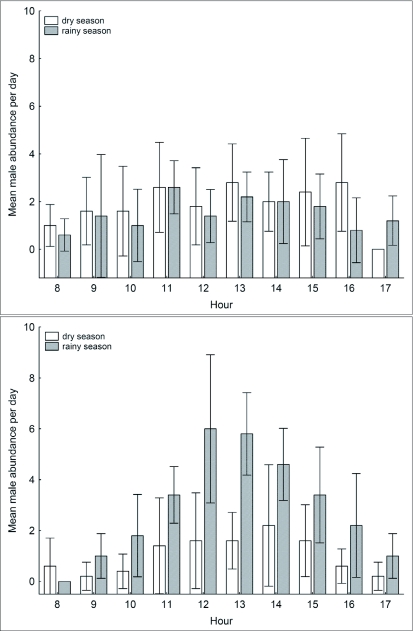
Variation in the abundance of *Paryphthimoides phronius* along forest edge transects taken hourly over the day on area 1 (A) and area 2 (B). Bars represent the 95% confidence interval for means of total count based on five sample days.

The variation in the daily abundance pattern of *P. phronius* differed from that of *H. hermes*, but was qualitatively similar between the two areas (Figure 3). In area 1, male abundance showed a small increase around midday in both seasons. In the dry season, male abundance remained stable until late afternoon, after which encounter rate decreased suddenly. The rainy season pattern was similar, although males were active even later, until after 1700 h. In area 2, *P. phronius* males peaked in abundance around 1200 h in the rainy season and around 1400 h in the dry season, followed by a gradual decrease until 1700 h. However, some males were still present after this time during the rainy season.

**Figure 4.**
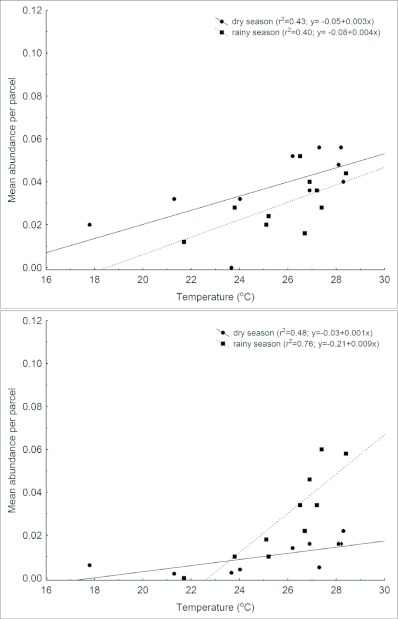
Relationship between mean male abundance of *Paryphthimoides phronius* and temperature in area 1 (A) and area 2 (B). Each point represents the temperature and male total counts averaged per hour across five sampling days.

**Figure 5.**
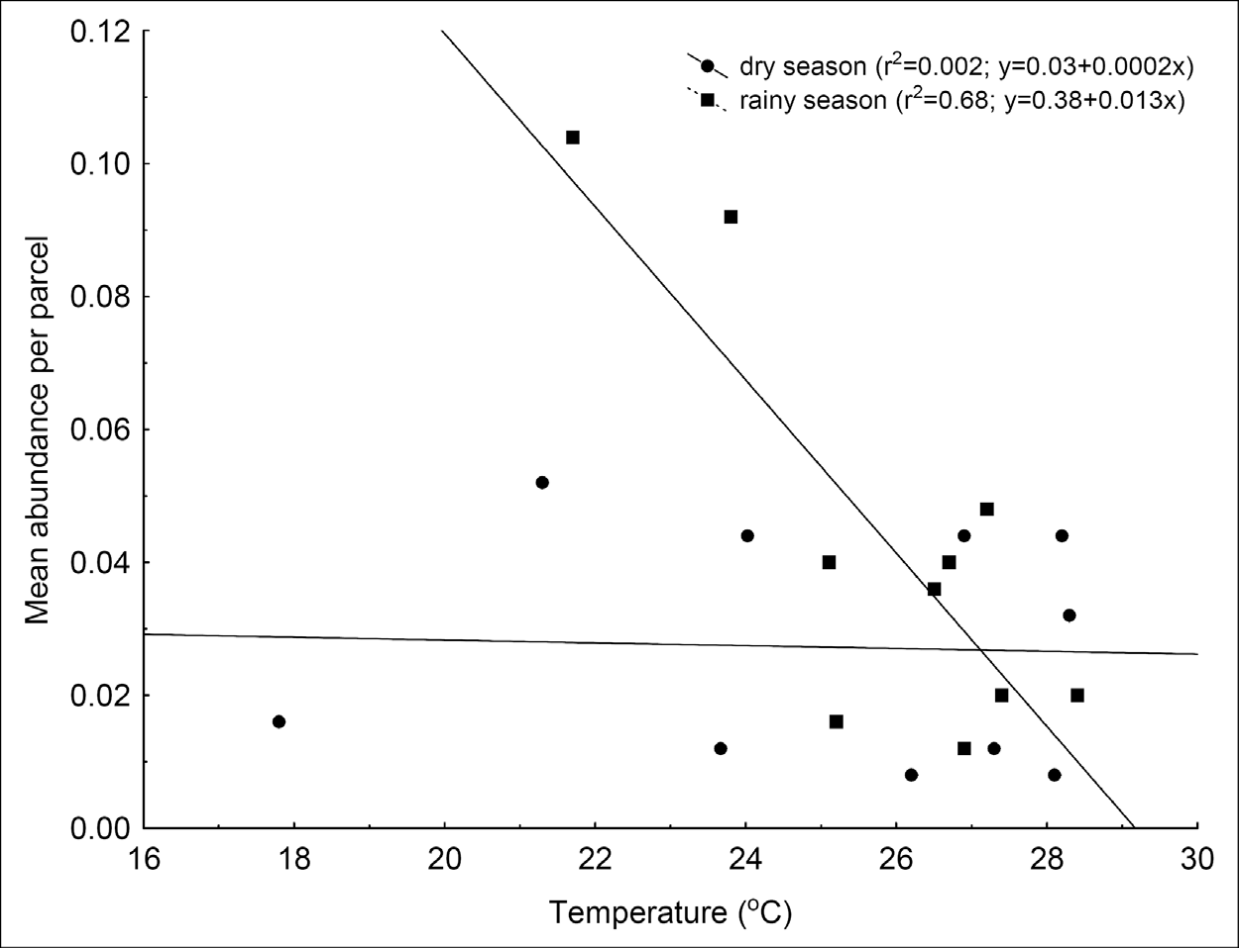
Relationship between mean abundance of *Hermeuptychia hermes* and temperature. Each point represents the temperature and butterfly total count averaged per hour across five sampling days.

In the area 1, the abundance of *P. phronius* males showed similar increasing rates with temperature in both seasons (Table 3; Figure 4A). In the area 2, the pattern was similar, although an interaction between season and temperature could be seen, with a higher increase in male abundance for each Celsius degree during the rainy season when compared to the dry season (ANCOVA_(temperature^*^season)_, F_(1,18)_ = 22.1, p < 0.001; Figure 4B). *Hermeuptychia hermes*, on the other hand, presented a very different pattern. In area 1 its abundance was not related to temperature variation in the dry season during the day, but in the wet season its abundance declined with increasing temperatures (ANCOVA_(temperature^*^season)_, F_(1,18)_ = 12.2, p = 0.003, Figure 5).

**Table 3.**
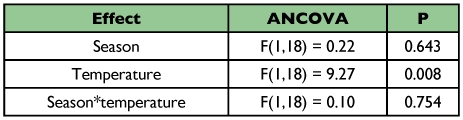
Covariance analyses showing the relationship between mean male abundance of *Paryphthimoides phronius* and temperature along the day and season in the area 1.

### Daily behavioral pattern of *P. phronius* males

**Figure 6.**
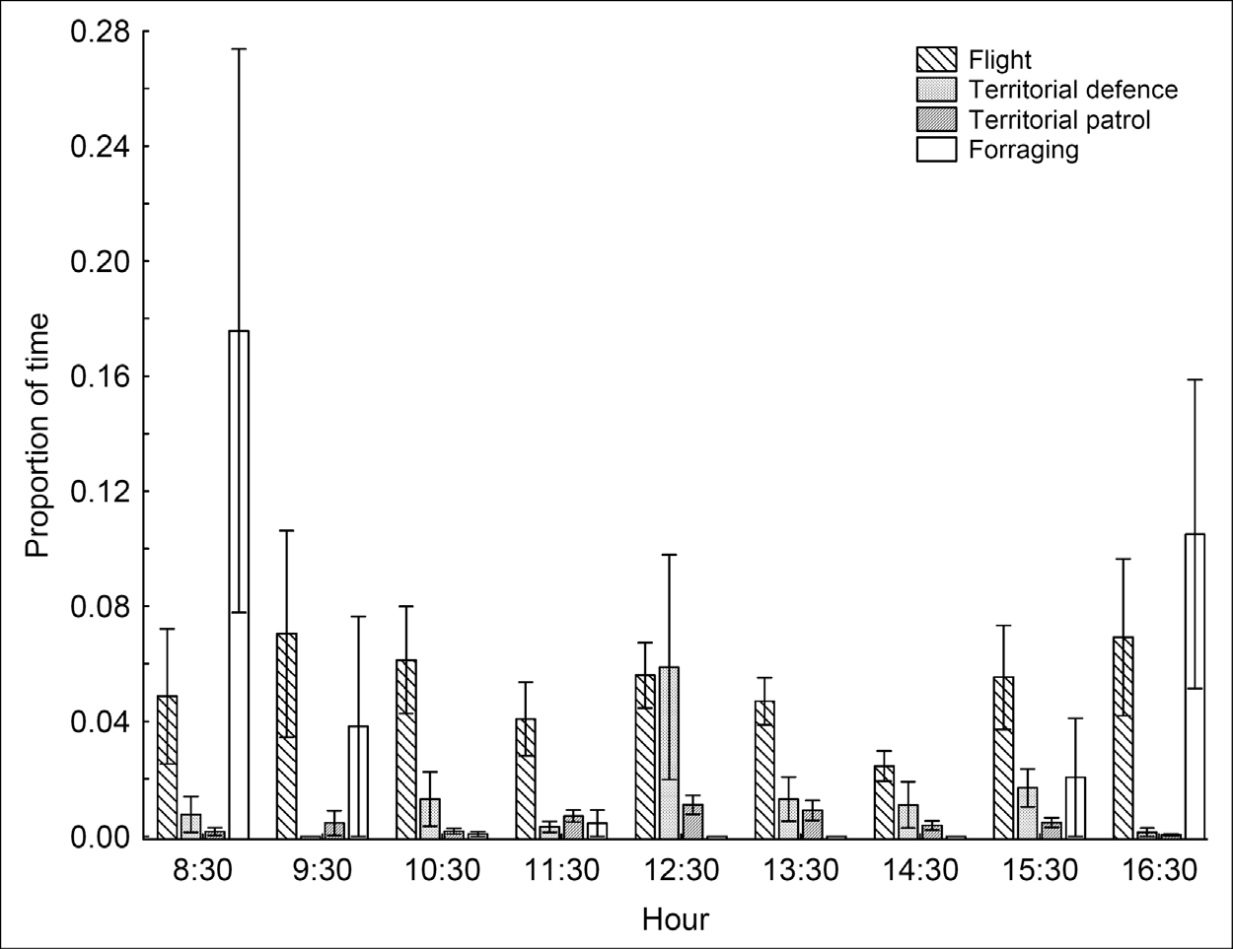
Daily activity budget of male *Paryphthimoides phronius* (based on 71 behavioral observations, 48 during the rainy season and 23 during the dry season). Each hourly estimate is based on means of five butterflies observed each on separate days. Bars represent standard error. Time spent perching is not shown.

The daily behavioral pattern calculated from focal observations on male *P. phronius* were variable, and the small sample size made it possible to recognize only stronger tendencies (Table 4; Figure 6). Butterfly feeding on decomposing fruit and grass was observed almost exclusively in the early morning and late afternoon. This pattern is in accordance with occasional observations made during the study. Males showed territorial behavior predominantly from 1100 to 1500 h. This behavior was evident both during the scans and the focal observations on males. Flights were observed all along the day at a rate of approximately 4–6%. The most frequent activity was perching, which occupied on average 88% (SE = 17%) of the male activity budget.

The territories of males *P. phronius* that engaged in territorial patrol and defense were generally located in sunny clearings near trees on the forest edge, although a few cases of males defending areas with decomposing fallen fruits were observed. The same territories tended to be used at the same sites on different survey days and by distinct territorial males that generally remained perched on the leaf litter or on leaves up to 50 cm from the ground.

Focal males that defended territories typically flew towards any butterfly that passed nearby (even much larger species). However, the disputes involving conspecific males were longer (mean = 22.6 s; SE = 6.47 s) than disputes between *P. phronius* males and other butterfly species (mean = 5.2 s; SE = 2.18 s) (Mann-Whitney test, U = 26, n_1_ = 14; n_2_ = 15, p < 0.001). Interactions between *P. phronius* males usually consisted of a series of relatively lengthy circular or spiral flights that ended when one of the butterflies left the area. These pursuits sometimes moved far away from the area that was commonly inspected by the territorial male. After a chase, both resident and intruder males could return and perch again in the territory before re-initiating the dispute. During the context, both rivals sometimes interrupted flight and, while perching on leaves inside the territory, often opened and closed their wings at regular intervals. Aerial disputes were typically conducted at high speeds, so that it was usually not possible to see details of the contest tactics of the contenders.

**Table 4.**
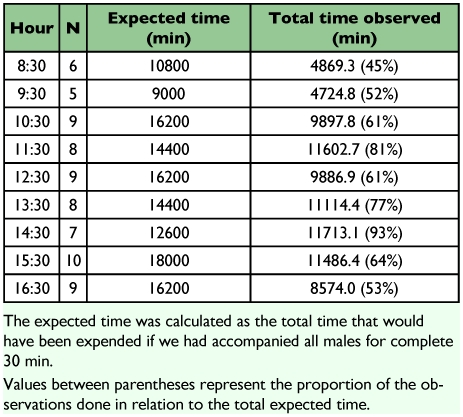
Number of observed males and total observation time for each hour during the daily activity pattern sampling (for wet and dry seasons) of *Paryphthimoides phronius*.

Although focal observations on *H. hermes* were not conducted, extensive observations on this butterfly at the study area provide no evidence of territorial behavior. Curiously, *H. hermes* defend territories in mid and late afternoon in other reserves (personal observations) using behaviors resembling those of *P. phronius*.

### Behavior of sexually receptive *P. phronius* females

**Figure 7.**
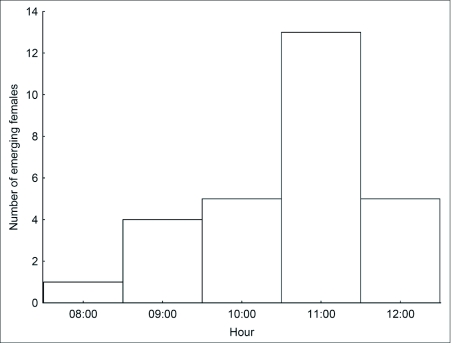
Number of lab-reared females emerging hourly from pupae.

Females raised in the laboratory emerged between 0850 h and 1250 h (n = 28; Figure 7). The majority emerged between 1100 and 1200 h, and only one emerged before 0900 h. The emergence time of males was not measured during the standard observations. However, six males observed in a pilot study emerged between 0950 and 1100 h.

The 14 females that emerged from pupa and were transported to the field on the day of emergence showed very similar behaviors. Freshly emerged females typically remained perched on or near the pupal skin for about 1 h until complete wing expansion (mean 56 min; range 31 – 95), and sometimes walked a short distance, before flying off. The behavior of 11 of these females was followed until they flew, seven of them for an additional hour after the first flight. None of these 11 females oriented to nearby males or to known mating territories, nor did they react to the presence of a male in the releasing areas. In general, after wing expansion, females typically exhibited many short flights until they flew toward the forest. Considering that the majority of females emerge from the pupa between 1100 and 1200 h and that they take approximately 56 min to expand their wings, it would be expected that they begin to fly when males are starting to defend territories.

Seven one-day-old, seven two-day-old, one three-day-old and one four-day-old lab-reared females were released near the males defending territories in areas 1 or 2 during the rainy season and after the beginning of male territorial defense. Six of the seven one-day-old females immediately flew up to the canopy and were lost from sight. The other one remained perched for 30 min before flying off. Four of the nine females two to four days old also flew directly to the canopy after releasing. Five remained perched for 1 h when observations were ended.

Two of the two-day-old unmated females were subsequently re-sighted near their releasing points; one of them was repeatedly observed at a place near decomposing fruits. Copulations involving two other females were observed among the 30 used in the release experiment. One involved the female that was released when it was four-days-old. This butterfly flew to the canopy after its release and approximately 5 min latter returned, entered a male territory (no decaying fruit was nearby) and was courted and mated. To start the copulation, the male flew in direction of the female until they perched and contacted their abdomens. After they remained in contact for 14 min, the mating pair separated and the female flew off. The other copulation involved a female that was released when it was three-day-old but was observed mating on the day following its release during the focal male observation period between male scans. The copulation started at 1330 h (after the arrival of territorial males in the area) near, but not inside, a territory commonly occupied by males. However, the mating male was not marked and its mating tactic (territorial or not) could not be identified before the copulation started. The mating was observed for 32 minutes until visual contact was lost.

## Discussion

These results show that two morphologically similar satyrinae butterflies that occur in the same area present different daily abundance patterns. *Hermeuptychia hermes* presented two abundance peaks over the day, while the abundance of *P. phronius* increased in the morning and remained stable during the afternoon. *Paryphthimoides phronius* abundance increased with temperature during both seasons, while *H. hermes* abundance was not affected during the dry season, but decreased during hot periods in the rainy season. Males of *P. phronius* started to defend territories in the late morning, while males of *H. hermes* were not seen adopting this behavior. Although females did not seem to be sexually receptive on the day they emerged, the time that males of *P. phronius* began to defend mating areas coincided with the time that newly emerged females began to fly. However, since the low numbers of newly-eclosed females released in the field was associated with difficulties in field observation, strong inferences on the post-eclosion female receptivity period cannot be made, and these conclusions must be treated with caution. In this sense, it seems interesting to note that another tropical butterfly Satyrinae species, *Hypolimnas bolina*, also needs approximately four days to become receptive (Kemp 2001). Although butterflies as a rule disappear from sight in the afternoon, the two small satyrines investigated here were active over essentially the entire daylight period.

One might expect that closely related species of similar appearance and habits should respond similarly to environmental conditions and adapt in similar ways when occurring together. Nevertheless, the differences in the general abundance patterns over the day between *H. hermes* and *P. phronius* suggest either that they have differently adapted to common physical conditions or that other biological differences in the two species take precedence. Similar differences in the daily activity pattern have been reported for small satyrine butterflies in tropical Australia (Braby 1995). According to this study, three sympatric species of *Micalesis* (Satyrinae: subtribe Mycalesina) tend to shift activities symmetrically around mid-day, with one species more active around mid-day and the other two with bimodal activity periods that peaks in the morning and again in the afternoon. A somewhat similar pattern occurred with two sympatric *Hypocysta* species (Satyrinae: subtribe Coenonymphina), in which one species peaked in activity shortly before mid-day and the other in the early afternoon.

The differences in the activity periods between *P. phronius* and *H. hermes* probably were a consequence of the conspicuous territorial behavior of the former species, making males visible along paths at the time males of *H. hermes* were dispersed in the brush. It seems relevant that the three species of *Micalesis* studied by Braby (1995) also separated into presumably non-territorial species that were active early and late (*M. perseus* and *M. sinus*) when the sun is low in the sky, and one reported to be territorial (*M. terminus*), which peaked in abundance during the midday period. The patterns reported for satyrine species in Australia and South America may be analogous. However, it is not clear why males defend territories on sunny areas when temperature is high. The territorial defense of areas with high light incidence such as sunspots or sunny clearings is common among butterfly species (Knapton 1985, Braby 1995, Dennis and Sparks 2005, Bergman et. al. 2007). In these situations, if females become receptive only in the afternoon and use these sites as landmark points to encounter sexual partners, males may be forced to occupy sunny areas, even during hot periods. On the other hand, the temporal and spatial separation seen in these satyrines is still little understood, and the possibility that activity differences are due to interspecific interference cannot be excluded.

The territorial behavior that we documented in *P. phronius* is similar to that reported for other territorial butterflies (Kemp and Wiklund 2001). Fixed mating territory sites are defended by resident males that return on successive days (personal observation). Resident males fly at a wide range of other insects, but lengthy combats only occur when the intruder is a conspecific male. Pursuits include linear, spinning and/or spiraling flights, and contests end with the departure of one individual. On the other hand, the territorial defense observed in *P. phronius* during both dry and rainy seasons is in contrast to other Satyrinae species, in which the occurrence of territoriality is seasonal (Wickman and Wiklund 1983; Alcock and O'Neill 1986; Ide 2002b). It is believed that the adoption of territorial defense in some species occurs in response to yearly variations of temperature or density. However, these studies are concentrated on temperate species that probably face great seasonal variations in density and temperature. In the present study, the mean temperature registered in the dry and wet seasons was very similar, a pattern that is expected to be very common in the tropical region. If temperatures do not show great seasonal variations, males of *P. phronius*, as well as other territorial butterfly species in the tropics, can likely function perfectly well at the high temperatures they encounter during hot summer and low temperatures from cool winter afternoons. Consequently, it may be postulated that any behavioral variation in tropical butterfly males may be preferentially related to female availability rather than to climatic conditions, although climatic conditions still can exert some influence.

Our limited observations on mating events suggest that the daily territorial tenure of *P. phronius* corresponds to the period in which receptive females become available for mating along forest edges. This type of relation makes sense if daily activity patterns are assumed to be adaptive, but few studies have actually sought a correspondence between the mate location schedule in males and female availability (Kemp and Rutowski 2001; Hirota et al. 2001; Ide 2004). However, in opposition to our initial expectation, the timing of female emergence from pupae is not causally related to their arrival at territories. Rather, females seem to require several days after emergence to become sexually receptive (about 4 days, if the data are representative), but when attaining maturity, these older receptive females may show up at territories around midday. It may be possible that, to help guarantee mating with a quality male, females should withhold mating with roving males (such as might occur in the morning) and seek mating exclusively in the afternoon with territorial males at ‘rendezvous’ sites (Kemp 2001). Kemp and Rutowski (2001) and Kemp (2001) report a seemingly similar situation for *H. bolina*. However, relatively few *P. phronius* were seen along edges in the morning, and our data do not exclude the possibility that, before moving onto territories, males may mate with females encountered during patrol searching, as was already reported for the butterfly *Lethe diana* (Ide 2004). Thus, it is possible that the afternoon territoriality in male *P. phronius* is at least in part due mate availability. However, why territoriality should occur in *P. phronius* and not in *H. hermes* is less clear.

Territoriality in butterflies is commonly adopted as mate acquisition tactic (Kemp and Wiklund 2001). The patterns observed in *P. phronius, H. hermes* and other tropical butterfly species studied so far (Braby 1995; Kemp and Rutowski 2001), suggest that even if climatic variations influences the male daily activity pattern, the need to find sexually receptive partners may represent a stronger determinant of male behaviors in the tropics. In this context, if females use specific encounter sites to mate, males may be forced to occupy sub-optimal places (i.e. sunny or hot areas) when temperatures are non-optimal. On the other hand, in species that do not need to use specific mating areas, males and females may respond to temperature variations in a more predictable way, occupying sunny areas when temperature is low and shaded areas during extremely hot periods. However, more data on tropical species, and especially, studies designed to simultaneously test the influence of female availability and climatic conditions on male behavior during the day, are necessary to test this possibility.
